# Natural air leak test without submergence for spontaneous pneumothorax

**DOI:** 10.1186/1749-8090-6-165

**Published:** 2011-12-24

**Authors:** Hidetaka Uramoto, Fumihiro Tanaka

**Affiliations:** 1 Second Department of Surgery, School of Medicine, University of Occupational and Environmental Health, Kitakyushu 807, Japan

**Keywords:** spontaneous pneumothorax, air leak, operation, VATS

## Abstract

**Background:**

Postoperative air leaks are frequent complications after surgery for a spontaneous pneumothorax (SP). We herein describe a new method to test for air leaks by using a transparent film and thoracic tube in a closed system.

**Method:**

Between 2005 and 2010, 35 patients underwent a novel method for evaluating air leaks without submergence, and their clinical records were retrospectively reviewed. The data on patient characteristics, surgical details, and perioperative outcomes were analyzed.

**Results:**

The differences in the clinical background and intraoperative factors did not reach a statistically significant level between the new and classical methods. The incidence of recurrence was also equivalent to the standard method. However, the length of the operation and drainage periods were significantly shorter in patients evaluated using the new method than the conventional method. Further, no postoperative complications were observed in patients evaluated using the new method.

**Conclusions:**

This simple technique is satisfactorily effective and does not result in any complications.

## Introduction

SP remains a significant health problem because the recurrence rate is estimated to be approximately 10%-20%, even after surgical management is performed [1]. Prolonged air leaks (PALs) are the main reason for prolonged thoracic tube drainage, a prolonged hospital stay, and increased costs [2]. We often experience postoperative air leaks in spite of there being no air leaks intraoperatively. Therefore, the procedure for evaluating air leaks itself might be a problem.

The submersion method, which is the most popular way of screening for air leaks [3], requires that the lung parenchyma be held to identify the leak point. Furthermore, most procedures are performed in a limited space by VATS. However, holding the lung puts it in a state that is far from its natural and physiological state. A practical technique that avoids this unnatural positioning has not been sufficiently discussed. We herein introduce a new technique that uses a transparent film and drainage tube. This method is easy and efficient, and does not appear to cause any complications. The purpose of this study was to determine the clinical outcome using the new method for detecting air leak

### Patients and methods

The institutional review board approved this study for the use of the data collection and analysis. From 2005 to 2010, two hundred twenty-one patients requiring surgery due to SP were included in this retrospective study conducted at the University of Occupational and Environmental Health. Preoperative investigations included chest radiography and a high-resolution computed tomographic (CT) scan of the thorax. Six patients with ipsilateral lung cancer and 1 lymphangioleiomyomatosis (LAM) were excluded from the analyses. Finally, a total of 214 patients were included in the present series. Thirty-five patients underwent this simple test for air leaks, and their clinical records were retrospectively reviewed. The indications for surgical treatment of patients with SP included PAL, tension pneumothorax, the obvious existence of bulla lesions at first presentation, hemopneumothorax, the occupation of the patient, and ipsilateral or contralateral recurrences. Data were collected for all patients and included a detailed history, age, sex, and smoking habits, past history of episodes of pneumothorax, treatment modalities, and surgical details. Postoperative variables included the pleurodesis, postoperative complications, and the duration of chest drainage. The patients were discharged from the hospital after surgery. Follow-up data were collected via attendance at the outpatient department.

### Surgical technique and patient management

**Figure 1:**
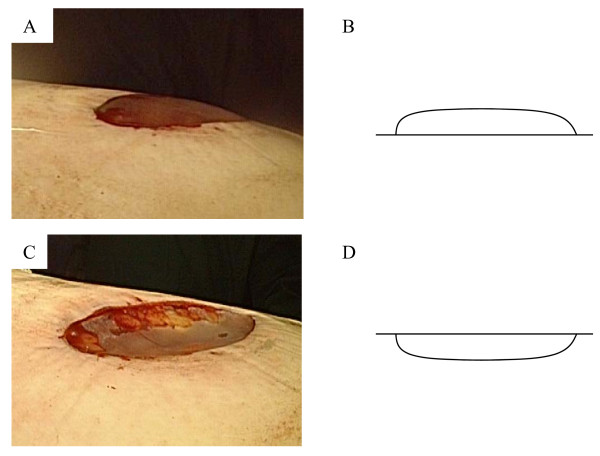
**A: An intraoperative photo shows a schematic diagram of the new technique**. If the covering film becomes convex this suggests that there is positive pressure in the thoracic space. B: A diagram showing the intraoperative view of (A). C: At the end of the procedure, if the transparent film has re-entered the inner side, this suggests the presence of negative pressure in the thoracic space (C). D: A diagram showing the intraoperative view of (C).

All patients underwent general anesthesia and single lung ventilation with a double-lumen endotracheal tube. The patients were placed in the lateral decubitus position. A 5 or 10 mm 0° video thoracoscope was inserted in the 7th intercostal space in the midaxillary line making use of the hole for the preoperative drainage tube. After inspection of the thoracic cavity, one or two additional ports were placed. The 5 or 10 mm working ports were placed in the 5th intercostal space between the scapula tip and the anterior axillary line. The blebs were grasped with an endograsp and excised with an endo-GIA stapling device (Auto Suture Company Division, United States Surgical Corporation, Norwalk, USA), ECHELON device (Ethicon Endo-Surgery, Inc; Cincinnati, Ohio) or were hand-stitched. One hundred ninety and 11 patients have undergone machine-mediated repair and hand stitching for blebs respectively. Both methods applied for 9 cases and 4 cases were performed only abrasion. The covering technique used was the (PGA) sheet (Neoveil) with fibrin-glue (Beriplast P; Marburg, Germany or Bolheal: The Chemo-Sero-Therapeutic Research Institute, Kumamoto, Japan). The new "natural" method that did not require submergence was as follows; the thoracic drainage tube was kept in the appropriate site through one of the port sites, and was not inserted into the interlobe after treatment of the bullous lesion. Next, the wounded area and port for drainage were temporarily closed by a transparent film (Tegaderm film 1626w 10 × 12 cm; 3 M Health Care, St. Paul. MN, USA), followed by connection between the drainage tube and thoracic bag (MERA Aquaseal D2; Senko Medical instrument Mfg, Co., ltd, Tokyo, Japan), and these were put in a low pressure suction machine (MS008-EX; Senko Medical instrument Mfg, Co., ltd). At the beginning of the surgical procedure, patients were ventilated at 15 cm H_2_O at the maximum inspiratory pressure and examined for air leaks. If the covered film became convex, this suggested the presence of positive pressure in the thoracic space (Figure 1A). Finally, patients were tested for the presence of air leaks up to a pressure of 20 cm H_2_O and negative suction of 5 cm H_2_O. If the transparent film re-entered the inner side, this suggested the presence of negative pressure in the thoracic space (Figure 1C). A mild change in body position was performed to clear the air at dead spaces as much as possible. If air leaks were found, a conventional intrapleural sealing test was carried out, and all air leaks were closed using sutures, staples or fibrin glue. A chest tube was placed if no air leakage was observed. On the other hand, the classical submergence method was to inflate the lung within a saline-filled pleural cavity held by a gauze ball [3]. The surgery was performed by a surgical team, including one surgical consultant, and procedures were selected based on the intraoperative situation.

### Postoperative management and follow-up

All patients were extubated in the operating room. The chest tube was connected to an aspiration system, and negative suction of 5-or 10 cmH_2_O was applied. Intercostal drains were removed when the underlying lung was expanded with no residual air leak. Postoperative pain was primarily controlled by means of a thoracic epidural block, oral analgesics were administered if necessary. Patients were discharged from the hospital when they were fully mobile. A follow-up was conducted for all patients. The median follow-up and relapse free periods were 134 and 207 days, respectively.

### Statistical analyses

Categorical variables were evaluated using the chi-square test and the t-test was utilized to analyze continuous variables between the two groups. Differences were considered to be statistically significant for p-values < 0.05. The data were analyzed using the Stat View software package (Abacus Concepts, Inc., Berkeley, CA).

## Results

In all, 214 patients were studied in this retrospective review. All of the patients were Japanese, including 182 males and 32 females in this series, with a mean age of 38.8 years (range 14-94 years). We compared the clinicopathological characteristics between the patients evaluated using the new (n = 35) and classical methods (n = 179). There were 35 (16.4%) total postoperative recurrences. There were no significant differences in the age, gender, smoking, lesion site, location, comorbidities, ipsilateral SP (ISP), contralateral SP (CSP), or surgery for ISP between the new and standard methods (Table 1). Furthermore, there were no significant differences in the approach, covering, or surgeon (Table 2). However, the length of the operation and drainage period were significantly shorter for patients evaluated using the new method than the conventional method. There were no significant differences in the perioperative outcomes, including pleurodesis, or postoperative complications and recurrence (Table 3). Further, no postoperative complications were observed in patients evaluated using the new method.

**Table 1:** The relationships between operation method and clinicopathological characteristics

Characteristic	Total	New	%	Classical	*p*-value
All cases	214	35	16.4	179	
Age (years)					
< 28	102	19	18.6	83	
≥ 28	112	18	16.1	94	0.391
Gender					
Male	182	31	17.0	151	
Female	32	4	12.5	28	0.523
Smoking					
Exist	75	11	14.7	64	
None	139	24	17.3	115	0.624
Lesion site					
Right	103	20	19.4	83	
Left	111	15	13.5	96	0.243
**Lesion location**					
Top	177	28	15.8	149	
**Other than top**	37	7	18.9	30	0.643
Comorbidities					
Yes	44	6	13.6	38	
No	170	29	17.1	141	0.584
ISP^a^					
Yes	50	10	20.0	40	
No	164	25	15.2	139	0.426
CSP^b^					
Yes	37	8	21.6	29	
No	177	27	15.3	150	0.341
Operation for ISP					
Yes	22	5	22.7	17	
No	192	30	15.6	162	0.394

^a ^ISP: ipsilateral spontenious pneumothorax, ^b ^CSP: contralateral spontenious pneumothorax.

**Table 2:** The relationships between operation method and intraoperative factors

Characteristic	Total	New	%	Classical	*p*-value
Approach					
VATS	189	32	16.9	157	
thoracotomy	25	3	12.0	22	0.531
Covering					
Use	173	28	16.2	145	
Non-use	41	7	17.1	34	0.890
Surgeon					
Consultant	103	13	12.6	90	
Trainee	111	22	19.8	89	0.155
Operation time (min)		99.4 (40-315)		130.2 (35-285)	0.046

**Table 3:** Periopetative outcomes

Characteristic	Total	New*	%	Classical	*p*-value
Pleurodesis					
Yes	15	0	0.0	15	
No	199	35	17.6	164	0.078
Postoperative complication					
Yes	15	0	0.0	15	
No	199	35	17.6	164	0.08
Postoperative recurrence					
Yes	35	7	20.0	28	
No	179	28	15.6	151	0.524
Drainage periods (day)		1.857 (1-3)		2.268 (1-9)	0.031

## Discussion

One of the most common complications after any procedure that involves the lung is PAL. Air leaks have also been shown to be more prevalent among patients with poor pulmonary function and among the elderly, who are making up an increasing number of such cases [2]. The only independent predictive factor for the recurrence of SP is the failure to identify and resect blebs during surgery [4]. The identification of blebs was formerly performed by a submersion method achieved by infiltrating the treated lung within the saline-filled pleural cavity [2]. This requires that the lung parenchyma be held to identify the leak point. Our postoperative recurrence rate after surgery was relatively high. These phenomena might be explained by the tendency to find more severe cases at university hospitals than in general clinics. We often experience postoperative air leaks in patients who did not have any air leaks during an operation. This is due, in part to the possibility of insufficient confirmation of the air leak points while undergoing surgery. It is desirable to test air leaks in the fully inflated lung because it represents a more natural way of measuring leaks. However, the working space of VATS is very narrow, which requires the lung parenchyma to be held in an unnatural state. In this situation, it is difficult to fully inflate the lung, making it more difficult to detect leaks. Thus, the surgeon may have to apply pressure to a partially front-and-center resected lung. However, this leads to both an incomplete evaluation, and a poorer evaluation of areas outside of the main lesion. These phenomena suggest that this test is inadequate. Therefore, we hypothesized that the evaluation of postoperative air leaks might be more effective when the residual lung is examined under a normal physiological state. Therefore, the submersion test can no longer be expected to provide good results for subjects who have undergone radical lung expansion.

We herein reported the development of a new method to test air leaks by using a transparent film and thoracic tube in a closed system. We believe that there are ten potential advantages associated with this new technique, without any apparent disadvantages. (1) The length of the operation and drainage periods were significantly shorter in patients evaluated using the new compared to the conventional method. This result can provide major benefits for the patients because it allows for minimally-invasive surgery and decreases the cost of medical care. (2) This method is performed in a natural and physiological state (3) It is easy to perform. (4) It takes just a few minutes to perform. (5) The new method does not require saline, which might cause pneumonia due to suction into the ventilated lung (6) Taking up the transparent film after sealing can clean up excrescent clots of blood, coagulation, and necrotic material, decreasing the risk of surgical site infection (7). This technique might be useful not only for SP, but also for limited resections such as wedge resection and segmentectomy for lung tumors (8). This method can be used for lungs treated *en bloc *rather than just from one region to the next. (9) This technique is inexpensive, and the transparent film costs just ¥308 ($3.76US). (10) Our technique is also accurate, is associated with very little risk, and will likely progress due to the expected advances in surgery in the future [5], especially since thoracoscopy has recently become popular, and most thoracic surgeons are already familiar with the procedure [6].

Nevertheless, our findings include several limitations for interpretation; primarily the retrospective nature of the study and the fact that it was carried out at a single institution. To overcome these limitations, prospective studies in a larger cohort of patients and at different institutions will be necessary to clarify the efficacy of this method. In conclusion, we herein presented a novel method for evaluating air leaks without submergence, decreasing the risk of unexpected air leaks after surgery.

## Abbreviations

SP: spontaneous pneumothorax; PAL: prolonged air leak; VATS: video-assisted thoracoscopic surgery; ISP: ipsilateral spontaneous pneumothorax; CSP: contralateral spontaneous pneumothorax; CT: computed tomography; LAM: lymphangioleiomyomatosis; PGA: polyglycolic acid.

## Competing interests

The authors declare that they have no competing interests.

## Authors' contributions

This report reflects the opinion of the authors and does not represent the official position of any institution or sponsor. The contributions of each of the authors were as follows:

HU were responsible for reviewing previous research, journal hand searching, and drafting report. FT was responsible for project coordination. All authors have read and approved the final manuscript.

## References

[B1] Muramatsu T Nishii T Takeshita S Ishimoto S Morooka H Shiono M Preventing recurrence of spontaneous pneumothorax after thoracoscopic surgery: a review of recent results Surg Today 2010 40 696 9 10.1007/s00595-009-4208-1 20676850

[B2] Abolhoda A Liu D Brooks A Burt M Prolonged air leak following radical upper lobectomy: an analysis of incidence and possible risk factors Chest 1998 113 1507 10 10.1378/chest.113.6.1507 9631785

[B3] Toloza EM Harpole DH Jr Intraoperative techniques to prevent air leaks Chest Surg Clin N Am 2002 12 489 505 10.1016/S1052-3359(02)00020-0 12469483

[B4] Naunheim KS Mack MJ Hazelrigg SR Ferguson MK Ferson PF Boley TM Landreneau RJ Safety and efficacy of video-assisted thoracic surgical techniques for the treatment of spontaneous pneumothorax J Thorac Cardiovasc Surg 1995 109 1198 203 10.1016/S0022-5223(95)70203-2 7776683

[B5] Uramoto H Takenoyama M Hanagiri T Simple prophylactic fixation for lung torsion Ann Thorac Surg 2010 90 2028 30 10.1016/j.athoracsur.2010.07.040 21095357

[B6] Oka S Uramoto H Hanagiri T Successful extirpation of thoracic pleural lipoma by single port thoracoscopic surgery Asian J Surg in press 10.1016/j.asjsur.2011.08.006 22208690

